# Editorial: Journey through bariatric surgery

**DOI:** 10.3389/fnut.2024.1546283

**Published:** 2025-01-07

**Authors:** Mauro Lombardo, Tyler McKechnie, Aristithes Doumouras, Edda Cava

**Affiliations:** ^1^Department for the Promotion of Human Science and Quality of Life, San Raffaele Open University, Rome, Italy; ^2^Division of General Surgery, Department of Surgery, McMaster University, Hamilton, ON, Canada; ^3^Clinical Nutrition and Dietetics, San Camillo Forlanini Hospital, Rome, Italy

**Keywords:** clinical nutrition, bariatric surgery, multidisciplinary support, weight loss, surgical management

## Introduction

Obesity, a growing global epidemic, poses a significant threat to public health, negatively affecting quality of life, and increasing the risk of several chronic diseases (1). In this scenario, bariatric surgery (BS) has emerged as the most effective intervention for the management of severe obesity, providing metabolic benefits and reducing comorbidities (2). With an increasing number of surgeries performed each year, there is a need to develop a multidisciplinary approach, integrating psychological support, nutritional management, and continuous monitoring to optimize outcomes. The journey of patients scheduled for BS should be optimized with personalized and standardized pre-, intra-, and postoperative pathways that encompass these multidisciplinary supports, to ensure the best outcomes after surgery. During this special Frontiers in Nutrition Research Topic, we had several submissions pertaining to the Journey Through BS that we'd like to highlight.

## Bariatric surgery and comorbidity management: the importance of pre and post-operative support

Bariatric surgery provides a significant benefit for patients with severe obesity and related diseases, but unfortunately not all the patients achieve the desired weight loss. Moreover, patients may experience a clinically relevant weight regain following a period of initial weight loss (3). Candidates for BS should be admitted to a comprehensive clinical follow-up program pre-surgery and then followed for the years after. For those patients who can be defined as “poor responders,” a further step of treatment after BS may be needed, and pharmacotherapy could be the choice. As shown by Vinciguerra et al., Liraglutide, a glucagon-like peptide-1 receptor agonists, was effective in the treatment of “poor responders” to BS, showing benefits in reducing metabolic syndrome and improving body composition, highlighting how the integration of targeted drug therapies can enhance the effects of surgery in individuals with insufficient weight loss. In the future, newly developed anti-obesity medications (AOMs) could aid in managing obesity and may be used as useful adjuncts for patients who do not experience the desired weight loss following BS.

In parallel, the role of vitamin D as a preoperative biomarker has proven crucial for the prevention of post-operative complications. The study from Shang et al. found that patients with low 25(OH)D levels had a higher risk of post-surgical readmission, indicating the importance of appropriate screening before surgery and vitamin D supplementation to reduce postoperative risks and improve recovery. The U-shaped relationship with 6-month readmission, suggests the importance of keeping the vitamin levels within the desirable range through individualized supplementation. Moreover, this study highlights the importance of a tailored choice to select patients and period of surgery, as shown by the surgery season related to 25(OH)D levels for the risk of readmission.

## Management of nutritional deficiencies and post-operative complications

Another example of the importance of considering vitamin deficiencies in patients undergoing BS is illustrated by a case of post-operative Wernicke-Korsakoff syndrome (Bento et al.). Pre-operative nutritional assessment and ongoing supplementation compliance are often underestimated after surgery, although they can lead to serious complications and generate potentially fatal neurological effects. It is evident that BS, although highly effective, requires rigorous nutritional follow-up to reduce the risk of complications and promote safe recovery with desired outcomes.

## The influence of psychological factors on surgical outcomes

In addition to clinical and nutritional screening, BS also requires a pre-operative psychological assessment. Indeed, outcomes depend not only on the surgical procedure, but also on the psychological wellbeing of patients, with elements such as anxiety, depression, and personality traits profoundly influencing the maintenance of long-term outcomes. A cluster analysis identified two distinct psychological profiles among BS candidates: patients with high levels of depression and eating disorder symptoms; both of whom tend to have greater difficulty in maintaining stable weight loss. Rodolico et al. suggest that targeted psychological support, could reduce the risk of failure and improve long-term outcomes. Taking these psychological factors into account during the preoperative evaluation is crucial for long-term success following BS.

## Lifestyles and long-term outcomes: an integrated approach

Another key to maintaining the benefits achieved after BS is a healthy lifestyle. Althumiri et al., stressed how a healthy lifestyle post-bariatric surgery is crucial for maintaining positive outcomes. This comparative analysis of dietary and lifestyle habits between patients living with and without obesity following BS found that a protein-rich diet and reduced smoking were strongly associated with better outcomes and a lower risk of weight regain. This analysis confirmed the importance of encouraging healthy lifestyle habits as part of an integrated care pathway.

## Inflammatory biomarkers and the role of Vanin-2

In the end, the dysfunction of excessive adipose tissue encountered in obesity is confirmed by increased inflammatory biomarkers, possibly contributing to the development of obesity associated comorbidities, such as type 2 diabetes (T2D), cardiovascular and cerebrovascular disease, among others.

Biomarker research in this field is burgeoning. Vanin-2 is a biomarker that was highlighted by Geng et al. in this Research Topic. Vanin-2 is a protein associated with inflammation of adipose tissue and insulin resistance, that has been shown also to be an effective predictor of BS outcomes. Geng et al. found that weight loss induced by sleeve gastrectomy resulted in a more significant decrease of Vanin-2 levels compared to weight loss after conventional dietary treatment. Therefore, monitoring Vanin-2 concentrations could help identify patients at risk of weight-loss failure, and to further customize post-operative management, with the ultimate goal of improving long-term outcomes.

## Conclusion: a multidisciplinary journey to success

BS is only one step in the journey of managing patients with obesity, which requires a multidisciplinary and personalized approach to address each patient's challenges. This Research Topic highlights the importance of an integrated holistic care, including psychological support, in-depth nutritional assessments and continuous monitoring of inflammatory biomarkers. BS, more than an isolated intervention, is a journey to a healthier life, in which every clinical and psychological aspect plays a key role in ensuring sustainable results and a better quality of life.
